# Rheological Behavior of Poly(Styrene-Co-Acrylonitrile)/Carbon Nanotube Sponges for Fiber Electrospinning Applications

**DOI:** 10.3390/nano15141060

**Published:** 2025-07-09

**Authors:** Rubén Caro-Briones, Marco Antonio Pérez-Castillo, Hugo Martínez-Gutiérrez, Emilio Muñoz-Sandoval, Gabriela Martínez-Mejía, Lazaro Ruiz-Virgen, Mónica Corea

**Affiliations:** 1 Escuela Superior de Ingeniería Química e Industrias Extractivas, Instituto Politécnico Nacional, Av. Luis Enrique Erro S/N, Unidad Profesional Adolfo López Mateos, Zacatenco, Alcaldía Gustavo A. Madero, Mexico City 07738, Mexico; rcaro@ipn.mx (R.C.-B.); mperezc1703@alumno.ipn.mx (M.A.P.-C.); gamartinezm@ipn.mx (G.M.-M.); lruizv1900@alumno.ipn.mx (L.R.-V.); 2 Escuela Superior de Ingeniería Mecánica y Eléctrica, Instituto Politécnico Nacional, Av. Luis Enrique Erro S/N, Unidad Profesional Adolfo López Mateos, Zacatenco, Alcaldía Gustavo A. Madero, Mexico City 07738, Mexico; 3 Centro de Nanociencias y Micro y Nanotecnologías, Instituto Politécnico Nacional, Av. Luis Enrique Erro S/N, Unidad Profesional Adolfo López Mateos, Zacatenco, Alcaldía Gustavo A. Madero, Mexico City 07738, Mexico; humartinez@ipn.mx; 4 Advanced Materials Division, IPICYT, Camino a la Presa San José 2055, Col Lomas 4a Sección, San Luis Potosi 78216, Mexico

**Keywords:** polymer composite solutions (PCSs), rheology analysis, CNT-sponges, electrospinning

## Abstract

Polymeric composite solutions (PCSs) reinforced with carbon nanotubes sponges (CNT-sponges) have attracted interest in material science and engineering due to their physicochemical properties. Understanding the influence of CNT-sponges content (0.1 wt.%, 0.3 wt.% and 0.5 wt.%) on rheological behavior of poly(styrene-co-acrylonitrile) P(S:AN) (0:100, 20:80, 40:60 and 50:50, wt.%:wt.%) solutions synthesized by emulsion polymerization can predict the viscoelastic parameters for their possible application in electrospinning processes. The obtained nanofibers can be used as sensors, textiles, purifying agents or artificial muscles and tissues. For this, amplitude and frequency sweeps were performed to measure the viscosity (*η*), storage (G’) and loss (G”) moduli and loss factor (tan δ). Most PCSs showed a shear thinning behavior over the viscosity range of 0.8 < *η*/Pa·s < 20. At low CNT-sponges concentration in the polymer matrix, the obtained loss factor indicated a liquid-like behavior, while as CNT-sponges content increases, the solid-like behavior predominated. Then, the polymeric solutions were successfully electrospun; however, some agglomerations were formed in materials containing 0.5 wt.% of CNT-sponges attributed to the interaction forces generated within the structure. Finally, the rheological analysis indicates that the PCS with a low percentage of CNT-sponges are highly suitable to be electrospun.

## 1. Introduction

Composite solutions [1] are materials made up of two or more compounds that are chemically and/or physically bonded. They have different characteristics such as high durability, hardness, toughness, fluidity, viscosity, flexibility and environmental degradation-resistance, as well as low density, corrosivity and greater electrical conductivity, among others [2,3,4,5,6,7]. These materials can have different proportions in their components and are separated by diverse interfaces [2,6,7]. This means that they are not soluble in each other, forming a synergy of final properties of composite materials [4]. The main constituents of composite materials are the matrix structures (continuous phase) that can be classified according to their metallic, ceramic or polymeric nature [6,8] and the reinforcement material in the form of fillers including particles, flakes, laminas or fibers which are dispersed, embedded and surrounded by the matrix phase, thus becoming a mostly complex system [2,3,4,6]. Thereby, the final physicochemical and rheological features of reinforced composites are related to concentration, distribution, homogeneity, orientation, size and shape of reinforcement [6].

Polymer–matrix composites (PMCs) can be modified by dispersing nanofillers such as metals, nanoparticles, nanofibers, nanorods, nanocrystals, nanodisks, nanoclays and nanotubes in the polymeric solutions [6,9,10,11,12,13,14]. Therefore, PMCs exhibit improved structural and functional characteristics compared to their corresponding unfilled counterparts [14]. Particularly, the addition of carbon nanotube sponges (CNT-sponges) into the PMC structure could affect the matrix properties and their rheological behavior in several ways, depending on their proportion, loading and possible orientation within the host matrix [14,15]. CNT-sponges are interconnected carbon nanotubes in helicoidal 3D networks [15], where their sp^2^-hybridized carbon bonding results in excellent chemical and thermal stability [15,16]. This is attributed to their stable skeleton, high porosity, elasticity, flexibility and light weight [16]. In addition, their chemical structure gives them good electron transfer kinetics, chemical inertness and enhanced conductive properties, which are useful in catalysis, tissue engineering, electronics, the sensors industry, drug targeting and energy storage [17,18,19,20].

Their elastic structure allows CNT-sponges to resist environmental changes, maintaining the shape and structural arrangement [15,16,20]. For example, they present a high tensile strength of 60 GPa, thermal stability from 600 °C up to 2800 °C, and Young’s modulus values close to 1.2 TPa [20]. These characteristics indicate that CNT-sponges show good elasticity, stress relaxation resistance and high degradation temperatures by the strong covalent bonds formed inside carbon nanotubes [15,17,20]. For this reason, their anisotropic geometry makes them appropriate for reinforcing polymeric matrices and turns them into oriented systems able to mimic natural structures [21,22]. Nevertheless, CNTs tend to agglomerate them, reducing parameters such as reinforcing efficiency and dispersion in a polymer matrix. This is caused by strong van der Waals forces between CNTs and their poor interaction with the polymeric matrix [16]. This behavior is often observed at high loadings of CNT-sponges (≥5 wt.%) [23].

It is worth mentioning that CNT-sponges display several advantages over traditional single-walled carbon nanotubes (SWCNT) and multi-walled carbon nanotubes (MWCNTs) [20,24]. This is explained by their unique helicoidal 3D structure, including enhanced rheological properties, high strength-to-weight ratio, flexibility, superior porosity, larger surface area (300–400 m^2^·g^−1^) and improved absorption capabilities. These properties offer versatile and practical solutions for a wide range of applications [20,24,25,26]. In contrast, while SWCNTs and MWCNTs possess high tensile strength and electrical conductivity; CNT-sponges are more difficult to disperse in solvents, or even need a functionalization process, for they can be used in a smaller variety of specific applications [20,24,25].

PCSs reinforced with CNT-sponges are often spun into fibers in different ways, including gel spinning and electrospinning techniques [22,27]. Some rheological properties are important parameters to fabricate fibers from these techniques and are related to the deformation of matter under applied stresses and changing the shear rates [28]. For this reason, the rheology properties can be an effective and efficient indicators to characterize the flow, homogeneity and dispersion properties of fillers into the polymer [28]. Through rheological measurements, the influence of fillers and mixing proportions on the fluidity can be effectively evaluated by means of the linear viscoelastic region (LVER) in the flow curves, relating to the viscosity, shear rate, yield stress, amplitude and angular frequency [28,29]. Further, a powerful tool for assessing the state of dispersion of the nanofillers in the polymeric matrix structure, as well as the filler–polymer interactions established at the interface, is the evaluation of the rheological behavior through the Han plot of PCSs, which shows a linear correlation in the plot of log storage (G’) versus log loss (G”) moduli [14,30,31].

Hence, the rheological parameters of PCS reveal fundamental information about the internal arrangement of reinforced polymers, allowing a complete understanding of the evolution of their structure and specific properties [14]. This viscoelastic behavior depends on various factors such as length, diameter, ratio, flexibility and volume fraction of fillers, as well as the concentration and the total solids content of polymers [32].

In this study, the rheological behavior of PCS was evaluated to determine if these materials are applicable in an electrospinning process for nanofibers formation. The rheological analysis revealed that the viscosity of materials reinforced with CNT-sponges ranged from 1 Pa·s to 20 Pa·s. Moreover, the PCS with a 0.1 wt.% and 0.3 wt.% CNT-sponges content showed a liquid-like behavior, and this indicates that the solutions are suitable to be electrospun. Otherwise, composites containing 0.5 wt.% of CNT-sponges presented a solid-like behavior attributed to their mostly elastic nature and the van der Waals forces generated, making them difficult to be used in the electrospinning technique. In brief, PCS with a low percentage of CNT-sponges have a better performance in producing nanofibers by the electrospinning technique.

## 2. Materials and Methods

### 2.1. Materials

The synthesis of poly(styrene-co-acrylonitrile) P(S:AN) solutions and carbon nanotubes sponges (CNT-sponges) were reported in previous works [33,34]. N,N-dimethylformamide (DMF) (Mw~73.09 g/mol) purchased from Sigma-Aldrich, St. Louis, MO, USA. Ethanol from Alquimia Mexicana, Mexico City, Mexico. Deionized water from Meyer, Mexico City, Mexico. All materials were used without further purification.

### 2.2. Carbon Nanotube Sponges Characterization

#### 2.2.1. X-Ray Diffraction (XRD) of Carbon Nanotube Sponges

The crystalline structure of CNT-sponges was identified by X-ray diffraction using a Bruker D8 Focus (Bruker, Madison, WI, USA), with high–intensity monochromatic Cu Kα radiation (λ = 1.541 Å), operating at 2 ≤ 2θ/° ≤ 120 at a scan rate of 2° min^−1^.

#### 2.2.2. Scanning Electron Microscopy (SEM) of Carbon Nanotubes Sponges

The morphology and structure of CNT-sponges were observed by SEM (JEOL JSM-7800F, Tokyo, Japan) at 10 kV and 2 kV using a working distance of 9.7 mm and 3.0 mm, respectively. For the SEM analysis, the CNT-sponges were dispersed in ethanol using an ultrasonic bath for 20 min. A drop of the prepared solution was deposited on a copper grid and dried using a visible light bulb at 25 °C. Statistical analysis measurements were performed manually using ImageJ ver. 1.52a (Research Services Branch, NIMH, Bethesda, MD, USA). Every SEM image was calibrated using the scale bar.

#### 2.2.3. Rheological Characterization of Carbon Nanotube Sponges

Rheological measurements of storage (G’) and loss (G”) moduli of CNT-sponges were made in a Modular Compact Rheometer MCR 502 (Anton Paar, Graz, Austria) using the parallel plate geometry with a 25 mm diameter on top, performing a shear strain γ = 1% and angular frequency (ω) range of 100 to 0.1 rad/s at 25 °C. All measurements were made by triplicate [33,35,36].

### 2.3. Preparation of P(S:AN)/Carbon Nanotube Sponges Solutions

The content of CNT-sponges in the PCS, P(S:AN), (0:100, 20:80, 40:60, and 50:50) (wt.%:wt.%) varied by 0.1 wt.%, 0.3 wt.% and 0.5 wt.% with respect to polymer weight. Samples of 0.001 g, 0.003 g and 0.005 g of CNT-sponges were ground in a mortar and pestle, then were weighed and well-dispersed by a sonication tip (Ultrasonic processor VCX500, Sonics & Materials, Newtown, CT, USA) in DMF for 2 h at 25 °C. Subsequently, 1 g of polymer was weighed and dissolved in the previous dispersion by mechanical stirring at 80 °C overnight. This protocol ensures the homogenization of solutions and evaporation of excess solvent.

### 2.4. Rheological Properties of P(S:AN)/Carbon Nanotubes Sponges Solutions

PCS were characterized rheologically as a function of CNT-sponges content and polymeric composition in a Modular Compact Rheometer MCR 502 (Anton Paar, Graz, Austria) using the parallel plate geometry (PP25) with a 25 mm diameter on top and a Peltier plate for temperature control on the bottom, with a 1 mm gap size between them. An evaporation blocker ring was used to avoid air disturbance during the measurements. For the first set of experiments, viscosity measurements were made by performing a shear rate ($\dot{\gamma }$) from 0.1 s^−1^ to 100 s^−1^ at 25 °C. In the second set of experiments, the linear viscoelastic range (LVER) was determined by performing an amplitude sweep with a steady angular frequency (ω) of 1 rad/s and a shear strain range (γ) 0.1–100% at 25 °C. To confirm the stability and homogeneity of polymeric composite solutions, a third set of experiments was performed (frequency sweep) by measuring the storage modulus (G’), loss modulus (G”) and loss factor (tan δ) at a shear strain within the LVER, γ = 1%, and an angular frequency range of 300 to 0.1 rad/s. To ensure that experiments are highly repeatable, all measurements were carried out in triplicate. [33,35,36].

### 2.5. Electrospinning Process

The PCS were placed in a 10 mL glass syringe (Dosys 155, SOCOREX, Ecublens, Switzerland), connected to a stainless-steel needle (21 G × 32 mm, Becton Dickinson, NJ, USA) through polytetrafluoroethylene (PTFE) tubing. The needle and the collector were separated with a work distance of 8 cm and connected as electrodes to a high-voltage power supply (ES30P-10W, Gamma High Voltage Research Inc., Ormond Beach, FL, USA). The applied voltage was set at 18 kV. Afterwards, the PCS were fed at 1 mL/h using a syringe pump (NE-1000, New Era Pump Systems Inc., Farmingdale, NY, USA) and finally, the polymer materials were electrospun until the fibers were obtained for 10 min at 25 °C.

#### Scanning Electron Microscopy (SEM) of Fibers

The morphology and diameter of fibers were observed by SEM (JEOL JSM-7800F, Tokyo, Japan) at 28 kV, using a working distance of 3.0 mm. To prepare samples, the fibers were dried at 60 °C and coated with thin gold layer by sputtering during 15 s. Statistical analysis from micrography was performed manually using ImageJ ver. 1.52a (Research Services Branch, NIMH, Bethesda, MD, USA). Every SEM image was calibrated using the scale bar.

## 3. Results and Discussion

Three series of PCS of poly(styrene-co-acrylonitrile) with different monomer ratios and several CNT-sponge contents were prepared. The codes and compositions of materials are summarized in Table 1. The PCS were prepared by direct sonication of CNT in DMF and mechanical stirring as described above. After preparation, black and homogeneous polymeric fluids were obtained. Additionally, the diameter, shape and crystalline structure of CNT-sponges were characterized before to be dispersed into the polymeric matrix.

### 3.1. Carbon Nanotubes Sponges Characterization Analysis

**Figure 1:**
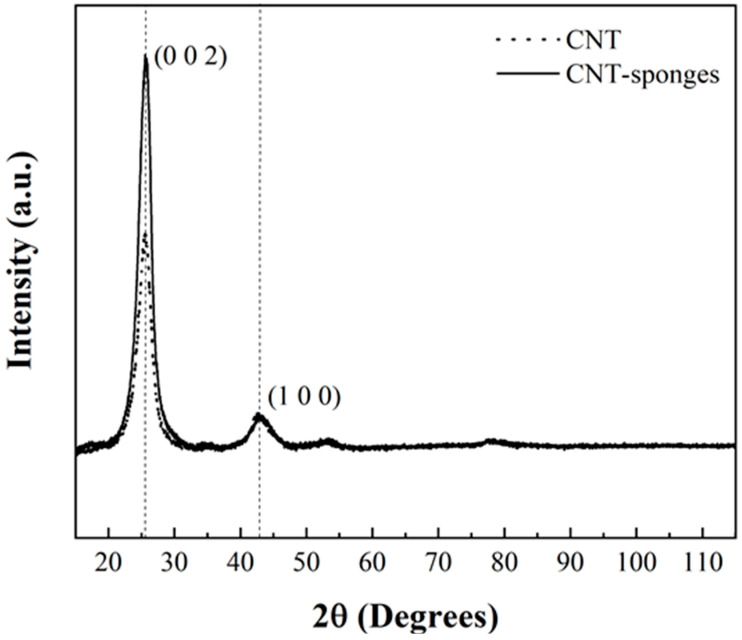
XRD pattern comparison for commercial CNTs and CNT-sponges.

The crystallographic structure of CNT-sponges was analyzed by powder X-ray diffraction. Simultaneously, Figure 1 shows the X-ray pattern of commercial multiwalled CNTs (Baytubes C150 P, Bayer Materials Science, Leverkusen, Germany) and the previously prepared CNT-sponges. It is observed that the diffraction pattern showed two main characteristic reflections at 25.85° (JCPDS: 96-101-1061) and 42.77° (JCPDS: 41-1487) corresponding to the graphite structure [37,38]. It is noticed that the intensity of the peak in the (002) plane indicates a higher crystallinity degree for the obtained CNT-sponges, almost twice the intensity reported for commercial CNT; meanwhile, the intensity of the peak in the (100) plane results to have the same intensity values.

**Figure 2:**
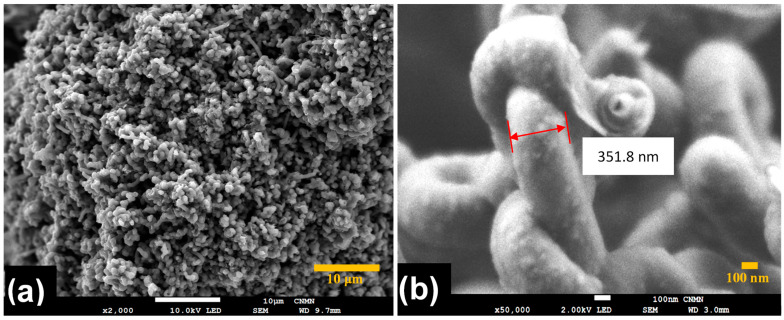
SEM micrographs of CNT-sponges under different magnifications: (**a**) ×2000 and (**b**) ×50,000.

**Figure 3:**
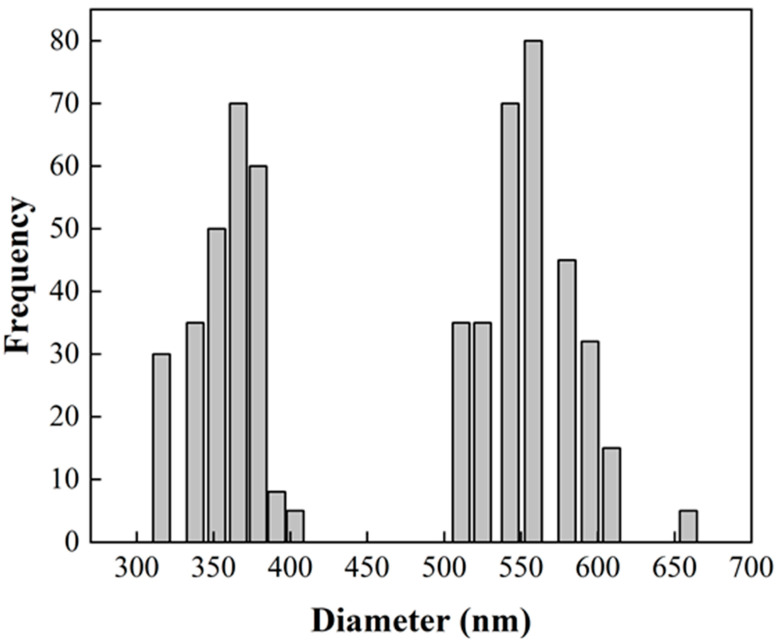
Statistical analysis of CNT diameter distribution from SEM image (Figure 2).

Morphology and size distributions of CNT-sponges were observed by SEM (Figure 2). The CNT-sponges show a tridimensional bamboo-like structure in Figure 2a. Some authors attribute this behavior to nitrogen atoms within graphitic lattices, which promotes the growth of additional carbon nanotubes in a zigzag pattern, leading to their entanglement and interconnection [32,37,38]. A statistical analysis of CNT diameter distribution was determined from SEM images. The obtained data were grouped into two classes, where the smallest diameters were 316.1 nm while the largest were 658.1 nm. After performing the statistical calculations for grouped data, average diameters of 557.6 ± 42.2 nm and 364.1 ± 29.6 nm were found for classes 1 and 2, respectively. As an example, Figure 2b shows CNT-sponges with a diameter of 351.8 nm belonging to class 2. The diameter distribution as a histogram is presented in Figure 3.

**Figure 4:**
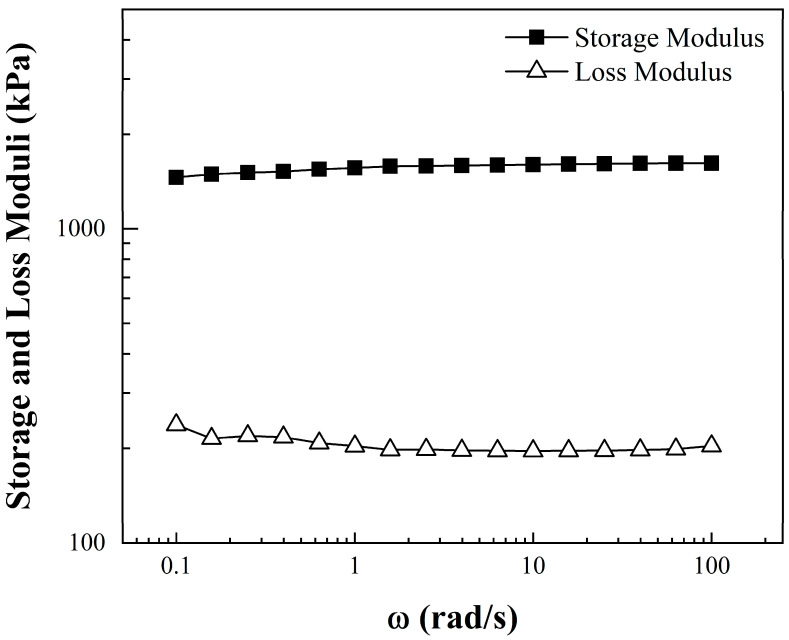
Storage and loss moduli as a function of the angular frequency (*ω*) of CNT-sponges.

Storage (G’) and loss (G”) modulus measurements were made to evaluate the elasticity of CNT-sponges structures. Figure 4 shows the viscoelastic moduli as a function of angular frequency (ω). Along with the entire frequency sweep, the storage modulus dominates and keeps a constant plateau at 1550 kPa ± 60 kPa above the parallel plateau corresponding to the loss modulus at 195 kPa ± 8 kPa. This is a clear behavior of a stable elastic/solid structure in the prepared sponges, where all the energy applied during the frequency sweep is stored by the tridimensional carbon nanotubes. Afterwards, when the sweep is retired, the energy is dissipated and CNT-sponges maintain their initial structure [39].

### 3.2. Rheological Behavior of Polymeric Composites Solutions

**Figure 5:**
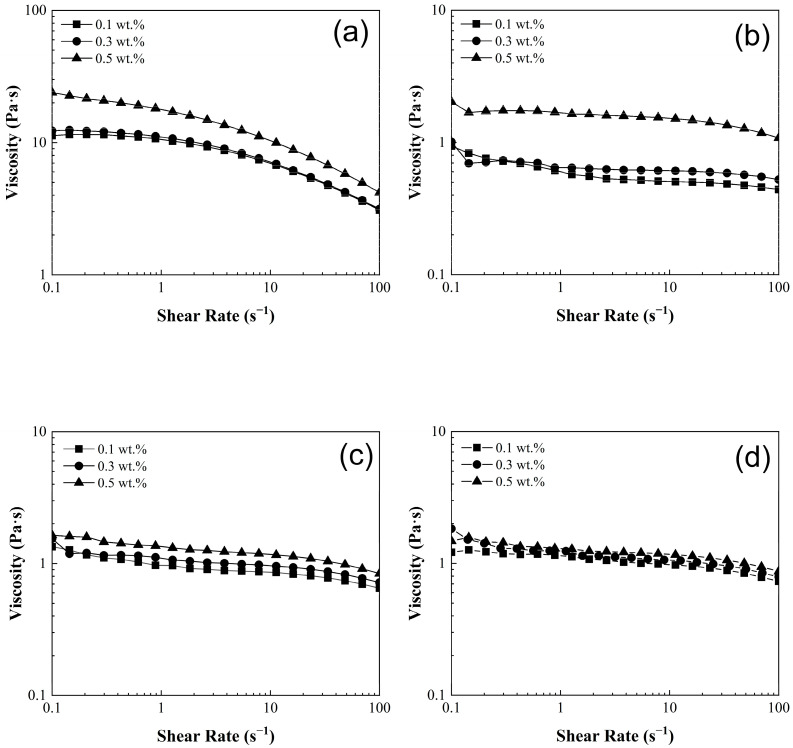
Viscosity (*η*) of poly(styrene-co-acrylonitrile) P(S:AN) solutions: (**a**) 0:100, (**b**) 20:80, (**c**) 40:60 and (**d**) 50:50 wt.%:wt.% with different CNT-sponges content.

Figure 5 shows the viscosity (*η*) of poly(styrene-co-acrylonitrile)/carbon nanotube sponge solutions at 25 °C. The viscosity results of the pristine polyacrylonitrile matrix (0:100, wt.%:wt.%) exhibited average values at 16 Pa·s ± 6 Pa·s (Figure 5a). It is noteworthy that in the pristine matrix, there is a transition from a viscosity plateau (Newtonian behavior) at low shear rates (0.1–1.0 s^−1^) to a shear-thinning region (non-Newtonian behavior) at higher shear rates (1.0–100 s^−1^). This smooth transition for PCS has been previously documented and is attributed to the re-alignment of carbon nanotubes. This phenomenon is commonly referred to as the rheological percolation of the CNT network within the polymeric matrix [14,40]. In contrast, the remaining composites solutions (20:80, 40:60, and 50:50, wt.%:wt.%) present significantly lower viscosity values up to 1.5 Pa·s ± 0.5 Pa·s (Figure 5b–d), nearly tenfold lower than those values exhibited by pristine matrix (0:100, wt.%:wt.%). Therefore, as the concentration of styrene increases in the polymeric solution, the viscosity decreases. In addition, these materials also reveal a consistent and gradual shear-thinning behavior across the entire shear rate range (0.1–100 s^−1^) as shown in Figure 5b–d.

According to the results presented in Figure 5, it is evident that there is a dependence between viscosity and the concentration of CNT-sponges in the polymeric solutions. This is more noticeable when CNT-sponge concentration rises within the polymer matrix. Analyzing once again the PCS, the polyacrylonitrile matrix (0: 100, wt.%:wt.%) presents approximate viscosity values of 10 ± 4 Pa·s at 0.1 wt.% and 0.3 wt.% of CNT-sponges while with increasing CNT-sponge proportion in the polymeric solution, the viscosity reaches values up to 20 ± 4 Pa·s at 0.5 wt.%. This behavior is attributed to three factors: (i) The saturation effect, where at low concentrations of CNTs, the viscosity is decreased. This is because there are fewer CNTs available in the system, so they interact less with the polymeric matrix. On the contrary, if the amount of CNTs increases, the viscosity rises. Nonetheless, as the CNT concentration becomes even higher, the viscosity does not change significantly. This is because a saturation point is reached, and the interaction sites are limited by the excess of CNTs. (ii) Dispersion and interaction of CNTs within the polymeric matrix. These play a crucial role. That is because at lower concentrations, it is usually easier to achieve a uniform and homogeneous dispersion of CNTs, leading to stronger interactions between individual nanotubes and the polymeric chains. However, as the proportion of CNTs increases, their dispersion in the system is more difficult and therefore the CNTs do not contribute to the increase in viscosity. (iii) Polymer–CNT interaction. The type, proportion and the affinity of a polymer towards carbon nanotubes are reflected in secondary (non-covalent) interactions generated between them. Hence, a high affinity and concentration of polymers drastically change viscosity behavior by the increased occurance of non-covalent interactions [39,41].

**Figure 6:**
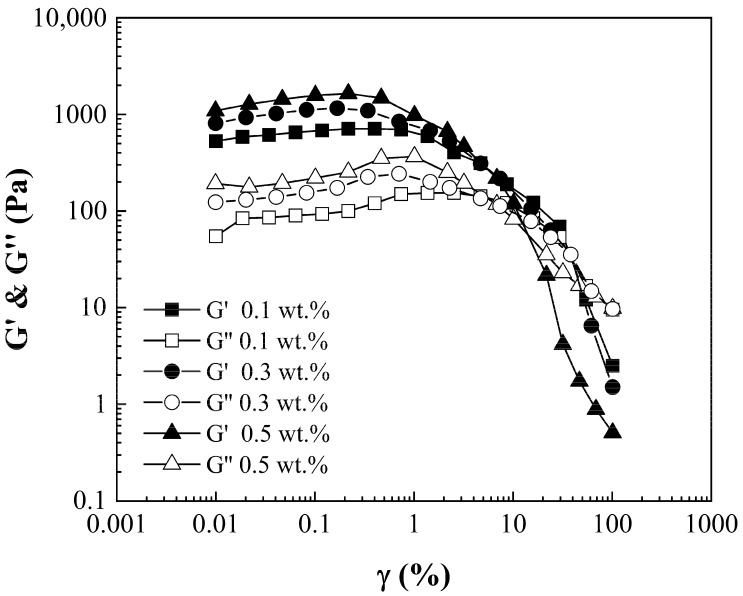
Amplitude sweep. G’ and G” moduli as a function of shear strain of poly(styrene-co-acrylonitrile) P(S:AN) solutions 0:100 with 10 wt.% of solids with different CNT-sponge contents.

Amplitude sweeps were conducted to indicate the LVER with a steady angular frequency of 1 rad/s. Figure 6 shows a plot for P(S:AN) 0:100 wt.%:wt.% at several concentrations of CNT-sponges as an example, because all PCSs have the same behavior. Storage and loss moduli show a LVER plate with a slight positive slope by the realignment of CNT-sponges into the polymeric matrix as shear stress is applied [28]. Elastic modulus values for 0.5 wt.% sponge contents were close to 1350 Pa·s ± 350 Pa·s for 0.3 wt.% up to 950 Pa·s ± 200 Pa·s, and 620 Pa·s ± 125 Pa·s, corresponding to 0.1 wt.%, denoting a rise in elastic modulus as CNT-sponges increased. Also, it is possible to visualize the limit of LVER, defined by the yield point (τγ), a key parameter that indicates the stress at which a material begins to deform plastically or flow. The values for the different CNT-sponges are *τ_γ_* (0.5 wt.%) = 0.51%, *τ_γ_* (0.3 wt.%) = 1.04% and *τ_γ_* (0.1 wt.%) = 1.48%.

**Figure 7:**
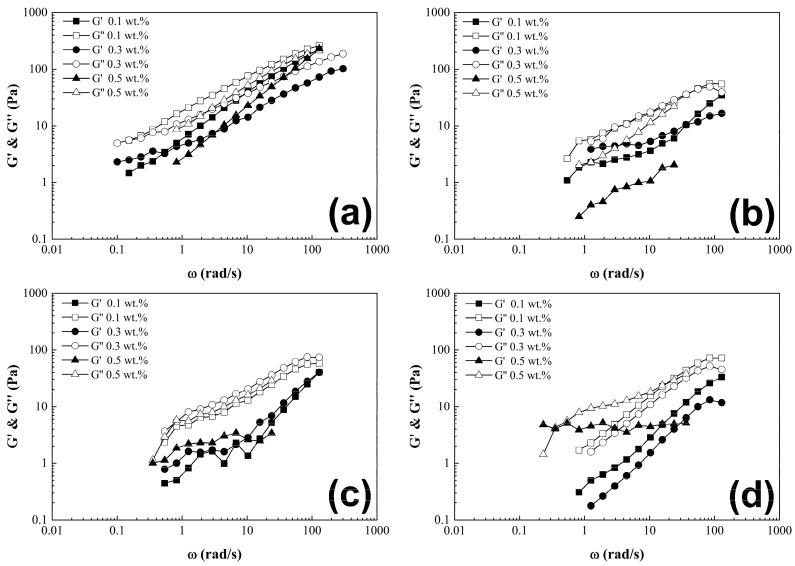
Frequency sweep. G’ and G” moduli as a function of angular frequency of poly(styrene-co-acrylonitrile) P(S:AN) solutions: (**a**) 0:100, (**b**) 20:80, (**c**) 40:60 and (**d**) 50:50 wt.%:wt.% with 10 wt.% of solids with different CNT-sponge contents.

Dynamic oscillatory experiments were conducted to evaluate the homogeneity of CNT-sponges within the polymer matrix. The strain applied should fall within the linear viscoelastic region of the material, in this case at *γ* = 1%, across a frequency range of 300–0.1 rad/s. Frequency sweeps for all PCS are presented in Figure 7. The G’ and G” modulus curves can describe the dispersion stability of CNT-sponge solution; if both curves present parallelism as long frequency sweep increases, this denotes a good dispersion, but on the other hand if curves have a different slope between them, or even a crossing point, this represents a lack of homogeneity or presence of agglomerations [14].

**Figure 8:**
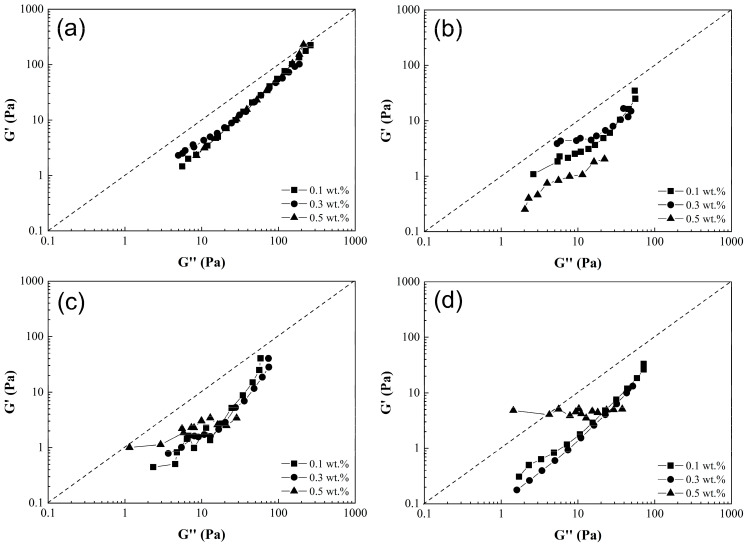
Log–log plot of (G’) as a function of (G”) of poly(styrene-co-acrylonitrile) P(S:AN) solutions: (**a**) 0:100, (**b**) 20:80, (**c**) 40:60 and (**d**) 50:50 wt.%:wt.% with 10 wt.% of solids and different CNT-sponge contents.

Figure 8 presents the curves of storage modulus (G’) as function of loss modulus (G”), commonly referred to as Han’s plot. This provides a quantitative assessment of solution homogeneity as the slope curve is positive, null or negative. In most polymeric composite solutions (PCS) shown in Figure 8a–d, a positive slope (value close to 1.37) was observed. This could indicate a uniform dispersion of CNT-sponges within the main polymer matrix. It is noticeable that the storage modulus increases proportionally to the loss modulus, and the yield point is at higher frequencies. Particularly, a null slope (horizontal line) was obtained for SAN5.4 as shown in Figure 8c,d. This null value could evidence the presence of CNT agglomerates, reducing the homogeneity of the solution [6].

Through further analysis using Han’s plot, it is possible to indicate the state behavior of polymeric composite solutions with a moduli relation over the entire frequency range. The G’ = G” ratio reveals a viscoelastic behavior of the material (denoted by a dashed line in the plots). Curves above this line (G’ > G”) indicate a solid-like state, while those below (G’ < G”) suggests a liquid-like state [42]. Initially, the solutions exhibit a liquid-like state, but with increasing frequency, their behavior approaches a viscoelastic-like state. The only instances of a solid-like state occur in solutions with a 0.5 wt.% of CNT-sponges (Figure 8c,d), according to the previously described analysis, attributed to the presence of agglomerations [43].

**Figure 9:**
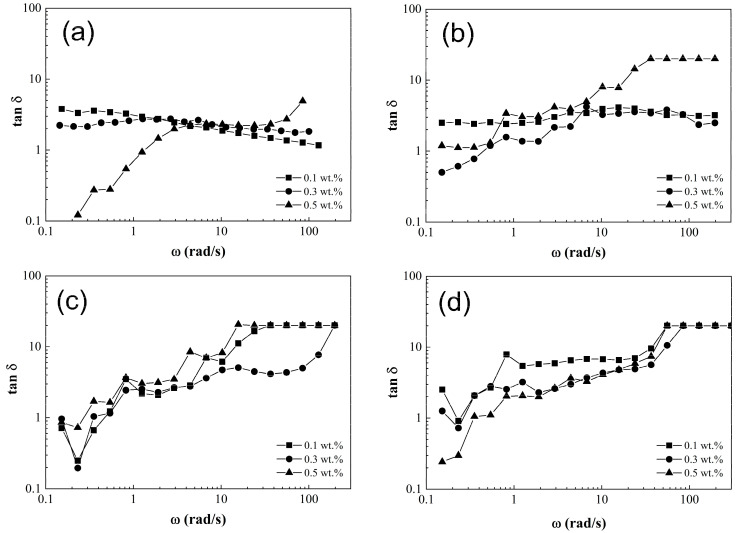
Loss factor (tan δ) as a function of frequency (ω) for poly(styrene-co-acrylonitrile) P(S:AN) solutions: (**a**) 0:100, (**b**) 20:80, (**c**) 40:60 and (**d**) 50: 50 wt.%: wt.% with 10 wt.% of solids and different CNT-sponge contents.

The loss factor (tan δ) is a parameter obtained from the frequency sweep that describes the PCS dissipation energy. This means, when tan δ < 1, the PCS presents a solid-like behavior; if tan *δ* = 1, it behaves as a viscoelastic material, but when tan *δ* > 1, a liquid-like behavior is found [39]. Figure 9 shows the tan *δ* (G’/G”) as a function of frequency (ω). It is observed that a pristine matrix with 0.1 wt.% and 0.3 wt.% of CNT content as shown in Figure 9a presents loss factor (tan *δ*) values above 1 for the entire frequency range, showing viscoelastic behavior. This is except for the polymeric composite solution with 0.5 wt.% of CNT which exhibits a mostly elastic behavior, because it undergoes a transition from a solid phase to a predominantly liquid phase [39]. Meanwhile, polymeric composite solutions (PCS) shown in Figure 9b–d reveal tan δ data have values under 1 at low frequencies (*ω*), but these values increase as long as *ω* is higher. This represents a transition from a solid-like to liquid-like behavior, attributed to the facility of CNT-sponges to be realigned and flow in the polymer matrix.

### 3.3. Fiber Morphology

**Figure 10:**
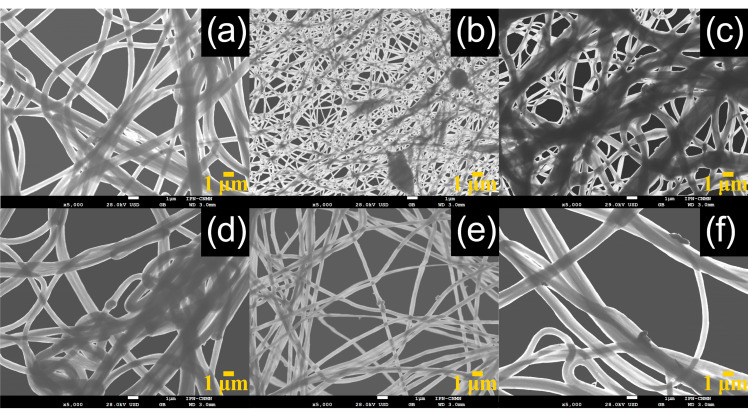
SEM micrographs of fibers at different P(S:AN) and CNT-sponges concentrations: (**a**) SAN1.1, (**b**) SA3.1, (**c**) SAN5.1, (**d**) SAN1.4, (**e**) SAN3.4 and (**f**) SAN5.4.

To observe the morphology and physical structure of reinforced fibers, all the materials were analyzed by SEM (Figure 10). The fibers were deposited, overlapped and entangled on a drum collector, obtaining a yarn-like structure.

**Figure 11:**
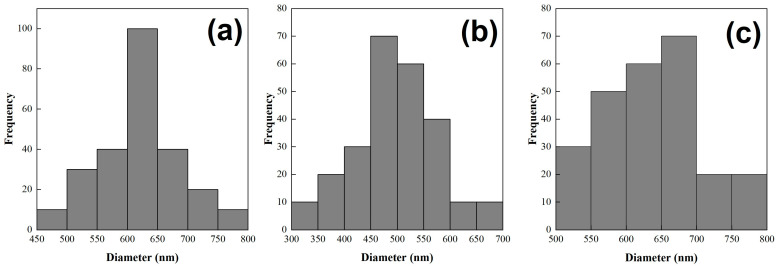
Statistical analysis of fiber diameter distribution with different CNT-sponges content: (**a**) 0.1 wt.%, (**b**) 0.3 wt.% and (**c**) 0.5 wt%; from SEM image (Figure 10).

In general, micrographs show a smooth and cylindrical morphology in the polymeric fibers with an average diameter of 650 nm ± 100 nm (Figure 10). A statistical analysis was carried out to measure the fiber diameter distribution as a function of CNT-sponges, and the histogram is presented in Figure 11. The mean value for three different CNT-sponge contents falls into the average diameter evaluated. However, diameter distribution is wider as CNT-sponge proportions increase. All fibers from the PCS were electrospun from PCSs within a viscosity range of 1.5 ≥ *η*/Pas ≥ 20, denotating that viscosity is an important indicator for processing homogenous fibers by electrospinning techniques. Reinforced fibers with a polymeric matrix 0:100 of P(S:AN) wt.%:wt.% present a structure free of agglomerations and protrusions (Figure 10a–c). However, composites with a polymeric matrix 50:50 of P(S:AN) wt.%:wt.% have agglomerations of CNT-sponges inside and/or attached outside of fibers (Figure 10b–d). This is because CNT does not show complete compatibility with polymeric solutions. These results agree with the rheological values obtained from the frequency sweep analysis, where the affinity between polymeric matrix and CNT-sponges plays a crucial role in the formation of fibers.

## 4. Conclusions

According to rheological results, it is possible to predict if polymeric composite solutions of poly(styrene-co-acrylonitrile)/carbon nanotube sponges will be suitable for electrospinning. First, it is necessary to use viscosity values within a range between 1 Pa·s and 20 Pa·s. If viscosity overpasses this range, possible segregations of polymeric matrix and reinforcement phases are obtained, producing agglomerations; hence, this will cause obstruction of the spinning needle, preventing the production of fibers or the modification of their morphology when the CNT-sponges are encapsulatied, as in the fringe case of dope SAN5.1 (*η* = 20 ± 4 Pa·s). On the other hand, if viscosity undergoes the electrospinnable range, the dope is electrosprayed, producing micro-nanodroplets of the polymeric solution, and no fibers are produced, as in the case of SAN1.2 and SAN 3.2 dopes (*η* = 0.8 ± 0.05 Pa·s). A second rheological study could define if the dopes present a viscoelastic, solid or liquid-like behavior. In addition, the loss factor (tan *δ*) for dopes with 0.1 wt.% and 0.3 wt.% of CNT-sponge content remained above one across the entire frequency range probed, presenting a liquid-like behavior acceptable for electrospinning. On the contrary, the dopes with 0.5 wt.% content presented a solid-like behavior, which made the dispersion more elastic and therefore hindering the electrospun process.

## Figures and Tables

**Table 1:** Contents of polymer and CNT-sponges in the polymeric composite solutions.

CNT-Sponges 0.1 wt.%		CNT-Sponges 0.3 wt.%		CNT-Sponges 0.5 wt.%	
Code	P(S:AN) ^1^	Code	P(S:AN) ^1^	Code	P(S:AN) ^1^
SAN1.1	0:100	SAN3.1	0:100	SAN5.1	0:100
SAN1.2	20:80	SAN3.2	20:80	SAN5.2	20:80
SAN1.3	40:60	SAN3.3	40:60	SAN5.3	40:60
SAN1.4	50:50	SAN3.4	50:50	SAN5.4	50:50

^1^ P(S:AN) wt.%:wt.%.

## Data Availability

The data presented in this study are openly available in the article.

## Abbreviations

The following abbreviations are used in this manuscript:

CNT	Carbon nanotubes
P(S:AN)	Poly(styrene-co-acrylonitrile)
PCS	Polymeric composite solution
PMC	Polymeric matrix composite
SEM	Scanning electron microscopy
LVER	Linear viscoelastic region
G’	Storage modulus
G”	Loss modulus
Τγ	Yield point
DMF	N,N-dimethylformamide
XRD	X-ray diffraction
PP	Parallel plate
PTFE	Polytetrafluoror
JCPDS	Joint committee on powder diffraction standards
